# The Evolving Role of Natural Compounds in the Medical Treatment of Uterine Fibroids

**DOI:** 10.3390/jcm9051479

**Published:** 2020-05-14

**Authors:** Michał Ciebiera, Mohamed Ali, Lillian Prince, Tia Jackson-Bey, Ihor Atabiekov, Stanisław Zgliczyński, Ayman Al-Hendy

**Affiliations:** 1 Second Department of Obstetrics and Gynecology, The Center of Postgraduate Medical Education, 01-809 Warsaw, Poland; michal.ciebiera@gmail.com; 2 Department of Surgery, University of Illinois at Chicago, Chicago, IL 60612, USA;mali78@uic.edu; 3 Clinical Pharmacy Department, Faculty of Pharmacy, Ain Shams University, 11566 Cairo, Egypt; 4 School of Public Health, University of Illinois at Chicago, Chicago, IL 60612, USA; lprinc4@uic.edu; 5 Division of Reproductive Endocrinology and Infertility, Department of Obstetrics and Gynecology, University of Illinois at Chicago, Chicago, IL 60612, USA; tjacks36@uic.edu; 6 Moscow Region Cancer Center, Balashikha 143900, Russian; i.atabiekov@gmail.com; 7 Department of Internal Diseases and Endocrinology, Central Teaching Clinical Hospital, Medical University of Warsaw, 02-097 Warsaw, Poland; stanislaw.zgliczynski@gmail.com

**Keywords:** uterine fibroid, leiomyoma, vitamin D, epigallocatechin gallate, natural, therapy, pharmacotherapy, phytotherapy, prevention, diet

## Abstract

Uterine fibroids (UFs) remain a significant health issue for many women, with a disproportionate impact on women of color, likely due to both genetic and environmental factors. The prevalence of UFs is estimated to be approximately 70% depending on population. UF-derived clinical symptoms include pelvic pain, excessive uterine bleeding, gastrointestinal and voiding problems, as well as impaired fertility. Nowadays numerous methods of UF treatment are available—from conservative treatment to invasive surgeries. Selecting an appropriate treatment option should be individualized and adjusted to the patient's expectations as much as possible. So far, the mainstay of treatment is surgery, but their negative impact of future fertility is clear. On the other hand, emerging new pharmaceutical options have significant adverse effects like liver function impairment, hot flashes, bone density loss, endometrial changes, and inability to attempt conception during treatment. Several natural compounds are found to help treat UFs and relieve their symptoms. In this review we summarize all the current available data about natural compounds that may be beneficial for patients with UFs, especially those who want to preserve their future fertility or have treatment while actively pursuing conception. Vitamin D, epigallocatechin gallate, berberine, curcumin, and others are being used as alternative UF treatments. Moreover, we propose the concept of using combined therapies of natural compounds on their own or combined with hormonal agents to manage UFs. There is a strong need for more human clinical trials involving these compounds before promoting widespread usage.

## 1. Introduction

Uterine fibroids (UF) are the most common benign gynecological tumor in premenopausal-age women, affecting up to 70% of women depending on the selected population [1]. In women of color this prevalence can be 80% [2]. Although considered benign, these lesions are often associated with significant morbidity. Up to 30% of afflicted patients become symptomatic with the most common manifestations: Abnormal uterine bleeding, pelvic pain, gastrointestinal issues, voiding problems, bulk symptoms, obstetric complications, and infertility [3]. Overall quality of life (QoL) in women with UFs is seriously impaired [4,5]. Recent studies from France found that about two thirds of surveyed women reported that UFs have a moderate to high impact on their QoL, highlighting the need for medical intervention [5]. About 30% of all hysterectomies among women of reproductive age are due to UFs [6]. Moreover, UFs are the most common indication for hysterectomy in the United States [7]. Surgical treatment choice for UFs imparts a substantial financial burden [8]. Cardozo et al reported that total annual cost of UFs in the United States, including direct and indirect costs, was estimated from $5.9 to $34.4 billion [9]. These data fully emphasize the growing awareness of the significant economic impact of UFs and urge the search for efficient alternative treatment as well as preventive options [10]. Observations made over the past years showed an increase in the number of women who wish to preserve their uteri for different reasons [7]. 

The careful selection of treatment modality should be made based on numerous criteria. Surgery remains the method of choice with the best cure rates; however, we can see a big move to minimally-invasive techniques through recent years. There is also an ongoing search to find efficient, cost effective, and safe anti-UFs drugs [7,11]. Recently, safety concerns have been raised for some drugs already available or under investigation [12]. Therefore, there is urgent need for alternatives to both prevent and treat UFs [13]. 

### 1.1. Uterine Fibroid Pathogenesis—Overview

Since the mechanisms underlying UFs pathogenesis have not been fully elucidated, numerous studies were and are still conducted in this regard. These mechanisms affect multiple levels of cellular and tissue function. Current UF origin theory believes that it is a mix of early life exposure (e.g., environmental) and estrogen hyper-responsiveness [14] with some additional factors that cause immunological changes [15] which result in impaired DNA repair leading to cell mutations [16]. Since 2011, with the presentations of results by Mäkinen et al. [17], most studies on UFs have focused on specific somatic mutations in the *MED12* gene encoding the mediator complex subunit 12 (*MED12*) [17,18]. According to available data, this mutation has been confirmed in more than 70% of patients with UF, depending on the population [17,19]. The UF life cycle might be divided into two steps: Transformation and tumor formation [18,20]. The transformation of normal myometrial stem cells into abnormal ones occur mostly through mentioned mutations. Myometrial stem cells transform into pathological ones and grow into uterine lesions mostly under the influence of hormones, and the growth of the tumors occurs through massive cell expansion and extracellular matrix (ECM) accumulation [21,22]. The ECM found in UFs is more abundant than in the healthy myometrium. It is believed that in here, the ECM volume may be over twice the volume of that found in the healthy myometrium. Various types of collagen, fibronectin, and proteoglycans are the main components of ECM [21,22]. Different fibers forming ECM found in UFs have an abnormal structure and differ from their counterparts in unchanged tissues [23]. 

Notably, ovarian sex hormones play a significant role in UF pathophysiology [18,24,25]. Recent data suggests that estrogen is important, but progesterone now is considered the key hormone initiating pathological differentiation and growth [18] and involves different pathways and growth factors [22,26]. Estrogen regulates transcription of proto-oncogenes and growth factor genes by binding to the nuclear estrogen receptor (ER)-α [27,28]. Andersen et al. found UF cells to be more responsive to 17β-estradiol than normal myometrial cells [29]. Dysregulation of steroid hormone receptors may be a primary pre-requisite for UF development [30]. Dose- and time-dependent induction of proliferation in UF cell lines in response to estrogen was also found in some laboratory studies [25,31]. Moreover, adenovirus-mediated delivery of a dominant-negative estrogen receptor gene abolishes estrogen- and progesterone-regulated gene expression in UF cells in vitro and shrinks UFs tumor in vivo [32,33]. Estradiol has a permissive effect on progesterone-mediated growth of UFs. Additionally, estrogens combined with progesterone significantly increase cellular expression of proliferation marker Ki-67 [34] and lead to ECM accumulation due to accelerated synthesis of collagen types 1 and 3 [35]. Ishikawa et al. demonstrated that UF xenografts have a 3-fold higher volume when treated with estrogen/progesterone combination versus estradiol alone or untreated controls, highlighting the significant role of progesterone in UF growth [24]. 

The action of steroid hormones on abnormal cells is mediated through different mechanisms, including a paracrine manner [18]. Cytokines and growth factors are large groups of protein mediators, transmitting information between cells that are responsible for differentiation, apoptosis, cell migration, and immunological mechanisms. As per UFs, they affect the growth and survival of their cells, regulate angiogenesis, and regulate the formation of the ECM [26]. Steroid-induced cytokines and growth factors affect UF signaling. This influences UF cells to grow and survive and ECM to accumulate. ECM is also some kind of reservoir for growth factors and cytokines as it increases their stability and extends their influence [21]. Among these growth factors, different isoforms of transforming growth factor (TGF-β) are one of the most important [22]. Laboratory studies have demonstrated that TGF-β reduces the concentrations of respective metalloproteinases (MMPs) and increases the concentration of their inhibitors (TIMPs) [36]. According to different studies, expression of TGF-β is significantly increased in myometrial cells that are in direct contact with the UF tumor [37]. TGF-β signaling is related to many pathways including Smad pathway, phosphoinositide 3-kinase (PI3K)/Akt/mammalian target of rapamycin (mTOR), the mitogen-activated protein kinases (MAPK) signaling cascade, and focal adhesion kinase (FAK) [22]. The other crucial factor in UF biology is activin A. It is produced by macrophages and it is responsible for different immunological actions including cell transformation, leading to tumor development [15,38]. Activin A is suggested also to be responsible for ECM production regulation [39].

**Figure 1:**
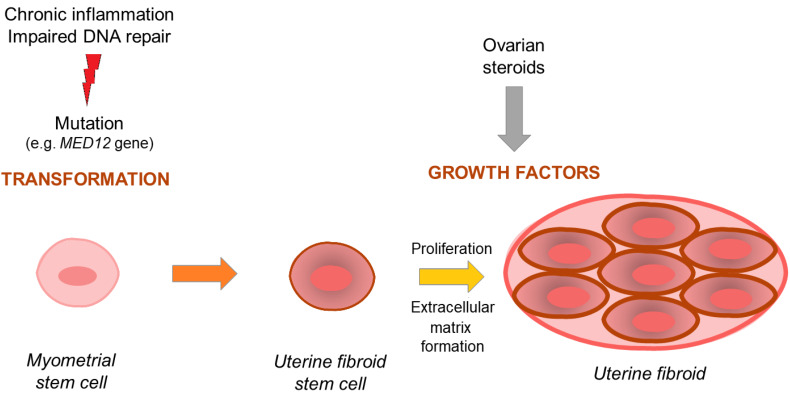
Development and growth of uterine fibroid—simplified scheme. Importance of mutation-derived transformation and stimulation of hormones and growth factors.

Wingless-type (Wnt)/β-catenin pathway is another important pathway in UF biology. It plays a role in somatic stem-cell function in the myometrium and in UF tissue [18]. It has been confirmed that β-catenin regulates and stimulates the renewal of stem cells [40]. According to recent studies β-catenin expression is increased in UFs compared to the adjacent myometrium samples [41]. Regulation of the biologic functions of β-catenin is highly complex. Wnt proteins bind to special cell-surface receptors, causing the activation of a cascade of proteins that leads to decreased β-catenin degradation in the cytosol which causes changes of β-catenin levels in the nucleus [18,42]. Ovarian steroids interact with the Wnt/β-catenin pathway to accelerate tumorigenesis [40]. In 2013 Ono et al. demonstrated that the paracrine activation of the Wnt/β-catenin pathway in UF stem cells promoted tumor growth [42]. It was then suggested that the canonical Wnt pathway may be a potential therapeutic target for the treatment of UFs [43]. Further research revealed that *MED12* gene silencing reduced the proliferation of UF cells and it was mediated by this canonical pathway [44]. A recent study by El Andaloussi et al. [45] demonstrated that *MED12* mutation presented a potential to transform cells by dysregulating Wnt4/β-catenin which affected mTOR signaling and caused autophagy abrogation, cell proliferation, and tumorigenesis [45]. In 2020, Ali et al. also found that β-catenin nuclear translocation contributes to UF phenotype, and β-catenin signaling is modulated by estradiol and histone deacetylases activity [46]. Additionally, the Wnt/β-catenin pathway leads to increased levels of TGF-β3 [18,22]. Some of those findings were supported with studies that used anti-UF agents which caused the attenuation of this pathway by reducing TGF-β3 signal and protein expression, resulting in a reduction in TGF-β canonical signaling [47]. In summary, interactions between Wnt/β-catenin and TGF-β pathways, as well as with steroids, give rise to the clonal formation of UF tumors and are believed to be basis of modern UF biology hypothesis [18,44]. The scheme of current UF origin theory is presented in Figure 1.

### 1.2. Uterine Fibroid Treatment Challenges

Surgical management via hysterectomy, myomectomy, uterine artery embolization (UAE), radiofrequency thermal ablation, and different types of focused ultrasound (FUS) remain as prime treatments for UFs. However, these are limited by financial burden as well as their impact on future fertility [3,48]. Pharmaceutical treatments have emerged like selective progesterone receptor modulators (SPRMs) and oral gonadotrophin-releasing hormone (GnRH) antagonist [49]. Injectable GnRH analogs are still limited as a short-course adjunct prior to surgery to improve anemia, as these cause osteoporosis, hot flashes, headaches, and other climacteric symptoms [49]. New oral GnRH antagonists (e.g., elagolix and relugolix) were found to be effective in reducing heavy menstrual bleeding in women with UFs and have a good safety profile [50]. Ulipristal acetate (UPA), one of the famous SPRMs, was reported to be highly effective in reducing UF volume, improving QoL, and reducing UF-related symptoms [51,52]. Until recently, they were drugs of choice for women who rejected surgery or who were prepared for a subsequent surgery [53,54]. However, UPA was found to cause different adverse effects. For example, progesterone blockade at the level of the myometrium could induce benign endometrial changes known as progesterone associated endometrial changes (PAEC), which required drug-free intervals to resolve. Such changes limit long-term use of these therapies [55]. Recent studies found that it could also cause liver failure that may require liver transplantation [56,57]. Clinical trials of vilaprisan, another SPRM, showed promising results. However, it was found to be potentially toxic in long-term research on animals and therefore all current trials were halted [58].

Given this body of evidence, there are many attempts to find an inexpensive, safe, long term, fertility friendly, and effective drug for the prevention and treatment of UFs [11]. Currently, only short-term usage and relatively high costs with questionable efficacy treatment options are available for UF medical therapy. The high amount of data in the literature suggests that natural and herbal compounds showed promising results on several types of tumors, suggesting that they may become future potential options for long-term UF treatment with minimal side effects. 

In this comprehensive review, we aim to summarize all published studies regarding using natural and botanical compounds against UFs in vitro, in vivo animal models, and in clinical trials.

## 2. Materials and Methods

Authors conducted their search on PubMed of the National Library of Medicine and Google Scholar. Databases were extensively searched for all original and review articles, as well as book chapters and published abstracts using keywords (single or in combination): Uterine fibroid; uterine leiomyoma; fibrosis; natural, botanical treatment; anthocyanin; berberine; collagenase, clostridium histolyticum; curcumin; docosahexaenoic acid; eicosapentaenoic acid; epigallocatechin gallate; fucoidan; indole-3-carbinol; isoliquiritigenin; lycopene; methyl jasmonate; resveratrol; quercetin; strawberry; sulforaphane; green tea; and vitamin D published in English until April 2020. Additional articles in bibliographies of reviewed articles were also searched. In summary, most relevant articles were reviewed and included as appropriate.

## 3. Discussion

### 3.1. Natural Compounds

In this section we will discuss the available natural anti-UF compounds in details.

#### 3.1.1. Vitamin D

Vitamin D is a common name for a group of fat-soluble steroid compounds that present pleiotropic effects on the human body with receptor found in various tissues [59]. Vitamin D fulfills all the requirements to be classified as a hormone [59]. Although it can be found in several forms, the most two described are ergocalciferol (vitamin D2 in plants) and cholecalciferol (vitamin D3-produced in the skin under the influence of sunlight) [60,61]. Studies have shown that cholecalciferol is more effective at raising serum 25-hydroxyvitamin D [25(OH)D] in patients with vitamin D deficiency as compared to ergocalciferol [60]. 

Vitamin D supplementation is usually recommended in oral or injectable forms [62]. The active form 1,25-dihydroxyvitamin D3 [1,25(OH)D] plays a central role in calcium–phosphate balance and regulation. Activation of vitamin D involves several steps in the body, starting with prohormone vitamin D synthesis in the skin, secondary to sunlight exposure, then conversion to 1,25(OH)D in the liver and kidneys [63]. Dietary sources of vitamin D include fatty fish like tuna, mackerel, and salmon, beef liver, egg yolks, and foods fortified with vitamin D such as dairy products, orange juice, and breakfast cereals [59].

Women of color have much higher risk of serum vitamin D deficiency compared to white women [18,19]. Up to 80% of non-Hispanic African American (AA) women had vitamin D deficiency versus only 20% of white women [64,65]. Vitamin D deficiency is an important risk factor for UFs development process and black women are also 3–4 times more likely than white women to have UF [3,66,67]. Given these parallel disparities, there is current interest to understand the role of vitamin D in the pathogenesis of UFs. Serum vitamin D levels are lower in women with UFs versus women without UFs [67,68]. Sabry et al. found an increased UF incidence in vitamin D deficient women and an inverse correlation between vitamin D levels and total UF volume [67]. Similarly, Baird et al. showed that the prevalence of UFs was found to be inversely related to vitamin D levels in 35‒49 years old African American and Caucasian females [69]. 

Vitamin D exhibited anti-proliferative, pro-apoptotic effects and induces cell differentiation in different diseases [70,71,72]. Vitamin D inhibits growth of UF cells through the down-regulation of kinases and Bcl2, and suppresses catechol-O-methyltransferase (COMT) expression and activity [73]. Vitamin D was tested on a rat model for UFs and a significant decrease in tumor size was observed [74]. Al-Hendy et al. demonstrated decreased estrogen-induced UF cell proliferation following vitamin D treatment [31]. Moreover, the expression of estrogen receptor (ER-α) and progesterone receptors (PR-A and PR-B) was inversely correlated to vitamin D receptor (VDR) expression in UF tissue compared to normal myometrium The same group reported that estrogen treatment decreased VDR expression and up-regulated PR-A and PR-B in UF cells [31]. On the other hand, vitamin D treatment significantly reduced the expression of ER-α, PR-A, PR-B, and steroid receptor co-activators (SRC) in vitro via induction of VDR expression. These effects indicate that vitamin D antagonizes sex hormones in UF cells and thus may have a role as an anti-UF treatment [31]. Similar results were showed using vitamin D analog paricalcitol both in vitro and in in vivo animal model of UFs [75]. 

The anti-fibrotic effect of vitamin D on UFs is further supported by reduction of transforming growth factor (TGF)-β3 induced ECM proteins expression as fibronectin and collagen type 1 in UF cells, which were found to be overexpressed in UFs [76]. As an important ECM modulating compound, vitamin D inhibits MMPs and decreases ECM production in UFs lesions, which correlates to UF phenotype and its bulk symptoms [77,78]. In 2018, Elhusseini et al. showed that vitamin D deficient diet in mice triggered low serum vitamin D levels and resulted in increased expression of steroid receptors in myometrium, increased expression of proliferation related genes, and enhanced inflammation and DNA damage [16]. Recently, Corachan et al. found that treatment with vitamin D did not change UF size when it was used for a short time; however, long-term usage induced a significant tumor volume reduction due to the lower cell proliferation rate in human-xenograft animal model [79].

**Figure 2:**
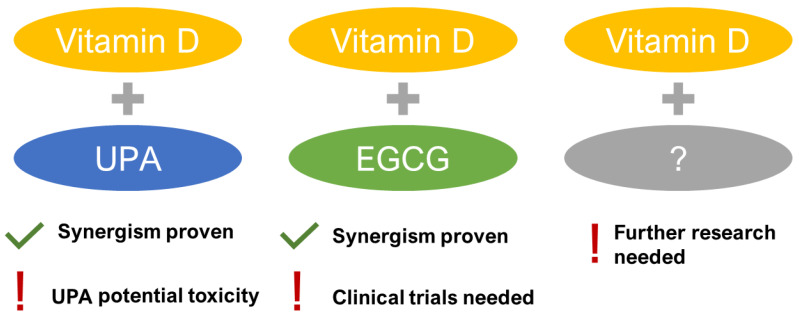
Synergistic combinations of Vitamin D in uterine fibroid (UF) therapy. EGCG: epigallocatechin gallate, UPA: ulipristal acetate.

Limited clinical trials are available. In 2016, Ciavattini et al. found that vitamin D-treated women presented with reduced disease progression in small UFs [80]. In a controlled study by Porcaro et al. in 2020, women with symptomatic UFs were treated with vitamin D together with epigallocatechin gallate (EGCG) and vitamin B6 for 4 months. The study found that total UF volume decreased by 34.7% in the treated group but increased by 6.9% in the control group. Authors concluded that such a combination might be a new form of non-hormonal treatment for women with UFs [81]. The concept of potential synergisms was also studied in the vitamin D and UPA combination with encouraging anti-UF effects both in UF cells [82] and in patients [83]. Both studies concluded that there might be a potential synergism between UPA and vitamin D to treat UFs [82,83]. However, the recent safety concerns of UPA induced liver injury ceased advancing the studies and consequently new synergisms are still to be explored (Figure 2).

Finally, a recent randomized controlled trial conducted in Iran by Arjeh et al. concluded that using 50,000 IU of vitamin D for 12 weeks did not change the volume of UFs but inhibited their further growth while the sizes of UFs in the placebo group increased [84]. 

Collectively, vitamin D has emerged as a notable candidate for UF treatment and possible prevention, considering its demonstrated anti-UFs effects along with a favorable safety profile. More human clinical trials are needed to confirm the effectiveness of vitamin D, alone or in combinations with other natural/pharmaceutical compounds, in UFs management.

#### 3.1.2. Epigallocatechin Gallate (EGCG) 

Green tea has been extensively studied for several health benefits. It is processed without fermentation, a key step in black tea production, resulting in its light color and high content of antioxidant catechin polyphenols [85,86]. There are five subtypes of these family including EGCG [87]. The single serving of green tea is estimated to contain up 150 mg of EGCG depending on the mixture [88]. Although catechins are typically consumed at nontoxic doses, adverse effects like abdominal discomfort, nausea, headache, and lightheadedness have been reported following daily doses exceeded 800 mg of EGCG for at least one month [89]. 

Studies showed that EGCG reduced inflammation and inhibits cell proliferation in various cancers [90,91]. EGCG effectively down-regulates cyclin dependent kinases (CDKs) such as CDK2 and CDK4 [92], induces apoptosis [93,94], and blocks angiogenesis and MMPs [95,96]—all processes that could prove beneficial outcome in preventing UF proliferation. Studies on UF animal model showed that EGCG effectively inhibited UF cells proliferation while induced cells apoptosis. EGCG significantly decreased the number and volumes of UF lesions in quails as compared to controls [97]. Several studies on rat ELT3 UF cells showed that high dose EGCG inhibited UF cell growth [86]. Treatment with EGCG led to 40% decrease in cell proliferation at 72 hours along with significant reduction of proliferation markers expression such as proliferating cell nuclear antigen (PCNA) and CDK4 proteins [86]. The same group found also that EGCG inhibits UF cell growth in a time- and dose-dependent manner. EGCG significantly inhibited cell growth after five days of exposure while a higher dose reached significance within 3 days after treatment [98]. In the same study, EGCG induced apoptosis with concentration-dependent downregulation of anti-apoptotic protein Bcl2 and upregulation of pro-apoptotic Bax [98]. Some genes of tumor suppressor gene p53 pathway were also upregulated in UF cells when treated with EGCG. Notably, EGCG induced apoptosis and decreased cell proliferation only in cancer but not normal cells, highlighting a favorable selective and safe effect [99]. Zhang et al observed a 14-fold increase of bone morphogenetic protein 2 (BMP2) expression in UF culture treated with EGCG compared to untreated control. BMP2 is a part of TGF-β family and plays a vast role in cell growth, division, differentiation, and programmed cell death [98]. Thus BMP2 can act as a pro-apoptotic gene. It has been previously investigated in epithelium of the human colon [100] where inactivation of BMP2 was shown to be associated with aggressive course of prostatic cancer [101]. Therefore, the ability of EGCG to increase BMP2 activity in tumors may lead to further investigating EGCG as an option for UF treatment.

COMT enzyme, which is involved in estrogen metabolism, is overexpressed in UFs versus normal myometrium [102,103]. Moreover, higher amounts of COMT are observed in black women compared to white [104]. EGCG has been shown to inhibit COMT, but there is a lack of data on human model [105].

In 2013, Roshdy et al. performed a clinical trial investigating the safety and efficacy of EGCG as anti-UF treatment. Thirty-three women with symptomatic UFs received 800 mg of EGCG daily for 4 months. The authors noted a significant decrease in UF volume, improvement in UF symptoms such as anemia, and increased health-related QoL scores as compared to controls, who reported symptom deterioration over the same period of time [106]. Recent study by Porcaro et al. [81], as we described in the vitamin D paragraph, showed a combined effect of vitamin D and EGCG with promising results.

EGCG analogs are also under extensive research. According to study by Ahmed et al. [107] the Pro-EGCG analogs 2a and 4a reduced susceptibility to COMT-mediated methylation and were found to inhibit proteasome and Akt signaling pathways. Authors concluded that these mechanisms might result in enhanced anti-proliferative, anti-angiogenic, and anti-fibrotic properties in UFs. EGCG appears to be a well-tolerated, readily available natural compound that presents promising results for treatment of UFs. Continued human clinical trials with larger cohorts and randomized control study design are essential to determine clinical effect of EGCG, alone or in combination, and measure its side effects. 

#### 3.1.3. Berberine

Berberine is a plant-based alkaloid used for centuries in Chinese medicine [108]. It can be found in *Scutellaria barbata*, a perennial herb which exhibited anti-inflammatory and anti-tumorigenic effects [109], as well as anti-proliferative, pro-apoptotic, and anti-metastatic actions [110,111]. Lee et al. showed that berberine-rich plants reduced the proliferative effect of human chorionic gonadotropin (HCG) in UF and myometrial cells wherein the expression of PCNA and cyclin E was significantly reduced under the influence of berberine [109]. The same authors also stated that *Scutalleria barbata* was potent in inhibiting intracellular aromatase in myometrial and UF cells [112]. Additionally, another study showed that berberine blocked estrogen- and progesterone-induced proliferation of UF cells as well as induced UF cell apoptosis, while not affecting human normal uterine smooth muscle cells [113]. 

Cyclooxygenase-2 (COX2) and pituitary tumor transforming gene 1 protein (PTTG1) were found to be more expressed in UF versus myometrium [114,115,116]. COX2 inhibition by celecoxib decreased the growth of UF cells, while hyper-stimulation of PTTG1 promoted cell growth significantly [114,115,116], suggesting the important role of both in UF pathogenesis. In 2017, Chuang et al reported that both COX2 and PTTG1 were significantly inhibited by berberine in UFs cells only. Briefly, authors performed an in vivo study using nude mice inoculated subcutaneously with rat ELT3 UF cells. Mice were then treated with 5 and 10 mg/kg doses of berberine, resulting in a weight reduction of the UF tumors by 60% and 85% respectively. Berberine significantly reduced the mRNA levels and proteins of COX2, PTTG1, Ki-67, PCNA, Cyclin D1, and CDK1 in dose-dependent manner [93]. No adverse events were observed in these mice, suggesting that berberine may be a safe and effective agent in UFs management. More investigations regarding berberine mechanism of action and safety profile are needed for expansion to human clinical trials.

#### 3.1.4. Curcumin

Curcumin is a yellow substance produced by some plants. It is used as food seasoning, cosmetic ingredient, or herbal supplement. Curcumin is the main natural polyphenol found in the rhizome of *Curcuma longa*. It has been traditionally used for decades in Asian countries as a medical herb due to its anti-microbial, anti-inflammatory, anti-tumorigenic, and anti-mutagenic properties [117]. According to the available data, curcumin might be applicable in UF therapy since it affected important UF-involved pathways [11]. It has been used in Chinese medicine formulations as a component of tumor-shrinking decoction with good effect [118]. Reports from Korea found that extracts from *Curcuma zedoaria* inhibited UF cell proliferation compared to normal myometrial cell as well as TGF-β receptor 2 in UF tissue [119]. Recently, curcumin was shown to inhibit TGF-β-related endothelial-to-mesenchymal transition and attenuated endothelial cell fibrosis [120].

In addition, Malik et al. showed that curcumin had an inhibitory effect on UF cell proliferation and ECM accumulation. It induced apoptosis [121] via regulation of caspase-3 and caspase-9, extracellular signal-regulated kinases (ERKs), and nuclear factor kappa-light-chain-enhancer of activated B cells (NF-κB) [121]. Similarly, Tsuiji et al. found that curcumin can decrease UF cell proliferation through the activation of peroxisome proliferator-activated receptor γ (PPARγ) and slowing down the processes in ECM [122]. Recently, Yu et al, using microarrays, found that *Curcumae rhizoma* in combination with *Sparganii rhizoma* inhibited the expression of UF cell proliferation and deposition of ECM-derived genes. Authors found that these agents were influencing the important UF-derived pathways including MAPK, PPAR, and TGF-β/Smad signaling [123]. Lately, it was found that curcumin presents powerful anti-inflammatory and anti-oxidant activities. It stabilizes the levels of interleukin 6 (IL-6), tumor necrosis factor α (TNF-α), ECM proteins (fibronectin and collagen), as well as a vascular endothelial growth factor (VEGF) [124].

In our opinion, data are not conclusive to recommend wide use of curcumin in UF therapy. However, it might be promising as a preventive treatment for women at high risk of developing UFs [121]. Notably, main challenges of curcumin are its low bioavailability and solubility. Yet new research about liposomal formulas showed promising results in this regard [125]. 

#### 3.1.5. Resveratrol

Resveratrol is a stilbenoid polyphenol that can be found in different plant species and also in red wines. Many studies demonstrated that resveratrol possesses a high antioxidant activity and anti-tumor activity [126]. Resveratrol can also be used in preventing lipid oxidation in pharmaceutical products, delaying toxic products formation [127]. Li et al. showed that resveratrol demonstrated apoptotic and anti-proliferative effects on human cervical cancer cells through the activation of caspase-3 and -9 and upregulation of Bax expression while downregulating Bcl-2 proteins [128]. Moreover, Resveratrol showed potent anti-inflammatory activity as lowered NF-κB, TNF-α, and IL-6 serum levels, as well as COX-2 activity and reactive oxygen species production in an animal model of acute pharyngitis [129].

*Scutellaria barbata*, which is source of berberine, also contains resveratrol, baicalin, apigenin, and luteolin. According to the study by Kim et al. on UF cells, extracted baicalin, berberine, and resveratrol did not inhibit UF growth, whereas apigenin and luteolin were effective. Authors concluded that compounds provided in *Scutellaria barbata* might stop the growth mediated by IGF-I in the human uterus, and may reduce tumor volume. However, the data about resveratrol was not evidenced [130]. Lately, studies have shown that resveratrol reduced the expression of ECM-related proteins like fibronectin, collagen types 1 and 3, as well as fibromodulin and biglycan. In the same study, resveratrol also reduced MMP-9 expression and increased tissue inhibitor of metalloproteinase 2 (TIMP2) protein expression in UF cells [131]. Moreover, Ho et al. stated that resveratrol inhibited UF cell proliferation through integrin αvβ3 pathway [132]. In the same study, resveratrol increased the expressions of pro-apoptotic genes p21 and inhibited the expression of anti-apoptotic genes. Authors also claimed it inhibited the Akt phosphorylation in UF cells [132]. Recently, data was confirmed by Chen et al. where resveratrol significantly suppressed UF growth in vivo via decreased expression of PCNA and α-smooth muscle actin (α-SMA). In addition, it decreased the mRNA levels of fibronectin, collagen type 1, and β-catenin. [133].

Collectively, these findings present a potential role of resveratrol in UF management. However, safety data is necessary to draw final conclusions. Also challenges regarding pharmaceutical compounding due to low solubility and bioavailability have been reported [126].

#### 3.1.6. Fucoidans

Fucoidans are a group of highly sulphated polysaccharides found in various species of brown seaweeds and brown algae. Fucoidans have been investigated for anti-oxidant, anti-inflammatory, anti-angiogenic, and anti-cancer activity [134]. The activity of fucoidans is related to their structure and depends on carbohydrate type, sulphate content, and molecular weight. Low molecular weight fucoidans were shown to have better cell cytotoxicity in comparison to native fucoidan in cancer cell lines, whereas gamma irradiated fucoidans presented with better cell transformation inhibition [134,135]. The role of fucoidans in fibrosis was highlighted by Li et al using mouse kidney model, where they were found to inhibit TGF-β1 induced epithelial–mesenchymal transition (EMT) and decrease expression of fibronectin and connective tissue growth factor (CTGF) [136]. A recent study by Wang et al. stated that fucoidans inhibited TGF-β1-related epithelial-mesenchymal transition through ERK pathway in lung [137]. Wu et al. [138] obtained the same data in radiation-induced fibrosis.

Regarding effects on UFs, Chen et al. [139] showed that fucoidan caused cell growth reduction, decreased fibronectin, vimentin, α-SMA, and the collagen protein levels. In this model, this natural compound reduced Smad2 and ERK1/2 pathways as well as β-catenin translocation. These data might suggest promising effects of fucoidans as anti-UF compound, however more basic research is necessary. 

#### 3.1.7. Indole-3-Carbinol

Indole-3-carbinol (I3C) is a common phytochemical compound found in cruciferous vegetables such as broccoli, Brussels sprouts, cabbage, cauliflower, and kale. Its biological activity on cellular and molecular pathways where tested mostly in cancers; however, I3C was also described as a blocking agent which upregulates drug-metabolizing enzymes [140]. According to current data, I3C has an influence on multiple signaling pathways and target molecules that regulate cell division, apoptosis, or angiogenesis in different known tumors, including UFs, such as PI3K/Akt/mTOR [140], as well as NF-κB [141], and promotes caspase 8-triggered apoptosis [142].

In 2020, Greco et al. showed that I3C and quercetin can regulate ECM expression, migration, and proliferation of primary UF cell. Authors found that I3C significantly decreased collagen 1 and fibronectin mRNA expression [143]. 

There are different clinical trials regarding the use of I3C in different cancer types and more work is still needed in research and clinical testing to start the usage of I3C for human malignancies [141]. More research is needed to support I3C as potential anti-UF agent.

#### 3.1.8. Isoliquiritigenin

Isoliquiritigenin is a phenolic compound found in plants that belong to licorice including *Glycyrrhiza uralensis*, *Mongolian glycyrrhiza*, *Glycyrrhiza glabra*, and others. This agent can also be found in different common foods and in alternative medicine. Isoliquiritigenin is a N-methyl-D-aspartate receptor (NMDA) receptor antagonist and a gamma aminobutyric acid (GABA) modulator and a metabolite [144]. Literature showed it might play an important role in immune response, Park et al. found that it inhibited NF-κB and interferon regulatory factor 3 activation [145]. Similarly, Feldman et al found that isoliquiritigenin has a potent influence on the inflammatory response of macrophages and inhibits the activation of NF-κB [146] and suppressed TNF-α-induced activation of adipocytes while activating peroxisome proliferator-activated receptor-γ [147]. Additionally, isoliquiritigenin was found to have an anti-tumor effect in melanoma [148] and anti-fibrotic effect in mesangial cells via inhibition of collagen, CTGF, and TGF-β1-smad signaling transduction [149].

Likewise in UFs, Kim et al found that UF cell proliferation was significantly reduced following isoliquiritigenin treatment in a dose-dependent manner with caspase-3 activation and Bcl-2 downregulation [150]. Other study by Lin et al found that isoliquiritigenin affected UF cell proliferation, apoptosis, and autophagy induction. Moreover, it arrested cell cycle and nucleus condensation only in UF but not myometrial cells. Isoliquiritigenin inhibited estrogen induced ERK1/2 activation as well as the expression ECM proteins and MMPs. Finally, it reduced serum levels of estradiol and progesterone levels in a mouse model [151]. These results advocate potential effect of isoliquiritigenin in treatment of UFs. However, clinical applications of this compound would be feasible after further studies.

#### 3.1.9. Quercetin

Quercetin is a plant flavonol with a bitter flavor that can be found in different fruits, vegetables, leaves, and grains. This compound is often used in beverages and foods. Usual daily consumption of quercetin equals of 25–50 milligrams [152]. Isolated quercetin also can be used by some people as a dietary supplement in daily doses of up to 1000 mg, however, this might be risky in some groups [153]. 

Quercetin polyphenolic substructure halts oxidation and act as a scavenger of free radicals [154]. Interestingly, quercetin has been reported to have estrogenic activities, it activates both types of estrogen receptor (α and β) and available data underline that it is rather β selective and weaker than estradiol [155]. Current studies highlighted anti-fibrotic effect of quercetin derivatives in hepatic stellate cells and showed a dose and time-dependent anti-proliferative effect. They reduced α-SMA and collagen I mRNA as well as reduced TIMP1 mRNA expression in TGFβ-induced cells [156]. In another recent study, quercetin presents with anti-fibrotic activity via influence on TGF-β/Akt/mTOR signaling pathway. In addition, it reduced IL-6, VEGF, and expression of different collagen types [157]. For UFs, in 2020 Cavalcante et al. found that quercetin showed uterine anti-aging features via PI3K/Akt/mTOR signaling pathway. The use of quercetin together with dasatinib was found to increase p53 gene expression and decrease *miR-34a* [158]. Moreover, quercetin and I3C significantly decreased collagen and fibronectin mRNA expression and the migration pattern in UFs [143].

Still, there is not much data about quercetin anti-UFs benefits, especially its estrogenic activity to affect tumor growth [159] as well as other sex steroid-dependent malignancies. More safety studies are needed since quercetin has been linked to nephrotoxicity [153].

#### 3.1.10. Sulforaphane

Sulforaphane is a natural agent that can be found in many cruciferous vegetables like broccoli, cabbage, cauliflower, and kale. It belongs to the isothiocyanate group of organosulfur compounds. Normally isothiocyanates are not present in the cells but are produced following cell wall destruction (e.g., by chewing). Sulforaphane is believed to play a role as a cytoprotective and chemopreventive agent [160], especially in carcinogenesis and fibrosis via alterations of the ECM [161]. For example, it was shown to protect the liver from fibrosis in an animal model [162]. A study explained fibrosis inhibition through modulation of the nuclear factor erythroid 2-related factor 2 (Nrf2) signaling that is involved in TGF-β signaling pathway [163]. It also attenuated pulmonary fibrosis by inhibiting the epithelial–mesenchymal transition, it decreased expression of N-cadherin, vimentin, and α-SMA [164]. Similarly, Fix et al found that sulforaphane inhibited TGF-β1 induced myofibroblast formation and reduces expression of integrins via inhibiting canonical and non-canonical TGF-β signaling pathways [161]. Another study confirmed the same findings in addition to decreased expression of various cytokines, TNF-α, and IL-6 [165].

There is not much data about the use of sulforaphane in UFs. However, recently Islam et al. presented their preliminary results where they found that sulforaphane inhibited UF cell proliferation and gene expression associated with inflammation and UF growth. Authors suggested that the use of this compound might find a place in the area of UFs [166]. However, more research is still required.

#### 3.1.11. Anthocyanins

Anthocyanins are water-soluble flavonoid pigments that may be present with red, purple, blue, or black color. Plants that are rich in those pigments include blueberries, raspberries or strawberries [167]. According to two studies, strawberries anthocyanin are of special interest in UFs [168,169,170]. Strawberries have anti-inflammatory, anti-oxidative, anti-proliferative, and genomic protective effects [169,171]. Islam et al. studied the effect of a variety of strawberry *Alba* cultivar extract on apoptosis, fibrosis, oxidation, and other processes in myometrium and UF cells. Authors found that anthocyanin-rich strawberries induced apoptosis and suppressed glycolysis and fibrosis in UFs cells as compared to control. Following strawberry treatment, authors observed an increase in reactive oxygen species levels in UFs. Additionally, anthocyanin-rich extract significantly reduced fibronectin, collagens, and versican mRNA expression in UF cells compared to untreated cells [168]. A recent study tested five different strawberry cultivars to identify which one presents the best phytochemical profile as an anti-UF agent. Authors found that *Alba* (same as in study above) and *Romina* cultivars presented the best results, with decreased collagen 1A1, fibronectin, versican, and activin A mRNA expression in UF cells [169]. Strawberry and its potent anthocyanins might offer therapeutic or preventive spectrum in UF treatment. However, further animal studies and then clinical trials are needed. These studies also emphasize that healthy diet rich in fruits like strawberries might be helpful for women with UFs.

#### 3.1.12. Omega-3 Fatty Acids 

Omega-3 fatty acids are polyunsaturated fatty acids (PUFAs). They are widely distributed in nature, being important in diet and having great impact on lipid metabolism. There are three major omega-3 fatty acids: Alpha-linolenic acid (ALA), eicosapentaenoic acid (EPA), and docosahexaenoic acid (DHA). ALA is found mainly in plant oils whereas DHA and EPA are common in fish and other seafood [172]. Humans, like other mammals, are not able to synthesize ALA and can only obtain it through diet. However, ALA can be transformed in humans to EPA and DHA by simple modifications. 

Omega-3 fatty acids play an important role in cellular function [173]. Despite many hypothesis and optimistic preliminary studies, dietary supplementation with omega-3 fatty acids did not affect the total risk of cardiovascular disease, cancer, or death [174]. Omega-3 fatty acids present with anti-inflammatory and immunomodulatory effects and might be potential therapeutic agents for inflammatory and autoimmune diseases [175] through decreasing the levels of C-reactive protein, IL-6, and TNF-α [176]. Since some of these pro-inflammatory molecules were found to be involved in the pathophysiology of UFs, omega-3 fatty acids were studied in that context. Islam et al. stated that myometrium has higher amount of arachidonic acid than UFs with ALA being higher in UFs. Treatment with EPA and DHA reduced the monounsaturated fatty acids content in UFs and controls. However, these did not reflect changes in the mRNA expression of ECM components. Omega-3 fatty acids reduced the levels of sterol regulatory molecules (e.g., ATP-binding cassette sub-family G member 1—ABCG1 or ATP-binding cassette transporter member 1—ABCA1) in both cell types. It also reduced cytochrome 450 family member—CYP11A1, the mitochondrial enzyme that catalyzes the conversion of cholesterol to pregnenolone. Authors concluded that omega-3 fatty acids modulate lipid profile, mechanical signaling, and cellular lipid accumulation in UFs [177]. Pregnenolone is a precursor for biosynthesis of many steroid hormones including progestogens, which is an important hormone in UF biology. This is an interesting line of thinking warranting further investigation.

#### 3.1.13. Methyl Jasmonate 

Methyl jasmonate is a natural derivative of jasmonic acid, which plays a role in plant defense, germination, growth, ripening, and senescence. Methyl jasmonate can induce the plant to produce anti-microbial phytoalexins, nicotine, or protease inhibitors [178,179] through interaction with NF-κB signaling pathways which is involved in various inflammatory cascades and regulate adaptive immune responses [180]. Accordingly, methyl jasmonate was tested and found to have a potent impact on cancer cells where it induced cytochrome C release in the mitochondria leading to cell death [181]. Methyl jasmonate showed antioxidant functions in adjuvant-induced arthritic rat models [182]. Umukoro et al. explained a membrane-stabilizing effect of methyl jasmonate [183]. Pereira-Marostica et al. in 2019 worked on brains of Holtzman rats with adjuvant-induced arthritis and found that it prevented arthritis-induced increased levels of nitrates, lipid peroxides, and reactive oxygen species [184]. A recent study by Ribera-Fonseca et al. highlighted the significant inhibition of gastric cancer cell migration and expression of different proteins related to the MAPK pathway in response to methyl jasmonate [185].

Methyl jasmonate inhibited enhancer of zeste homolog 2 (EZH2) activity which is linked with different tumors, including both benign, like UFs, and malignant types [186,187]. Ali et al. showed that methyl jasmonate demonstrated potent selective anti-proliferative effect on UF cells, even in low concentrations, as compared to myometrial cells. Moreover, methyl jasmonate decreased RNA levels of EZH2, collagen 1A1, fibronectin, cyclin D1, PCNA, and β-catenin, while increasing expression of p21, Bax, and cleaved caspase 3 in UF cells in comparison to untreated control [186]. This novel compound might be a potential anti-UF candidate warranting further investigation.

#### 3.1.14. Lycopene

Available data suggest a reduced risk of UFs among women with a greater dietary intake of fruit and vitamin A [188]. Lycopene is a phytonutrient of carotenoid family, defining the orange and red color of many widely consumed fruit and vegetables including tomatoes, carrots, papaya, and watermelon. Free radicals cause oxidative damage to tissues leading to disturbances in cell proliferation and differentiation. This results in DNA alterations potentially leading to cancer development [189,190]. Carotenoids are well-known antioxidants that protect cells from the above-mentioned processes [191,192,193,194]. Rao and Agarwal found that serum lycopene levels are low in patients with chronic illnesses and oxidative stress. In the same study, they have observed a significant decrease in thiobarbiturate-reactive substance, a marker of oxidative stress, in individuals supplemented with lycopene [195]. Many studies have shown that lycopene intake decreased the incidence of lung, prostatic, breast, cervical, and gastrointestinal cancers in humans [196,197,198,199]. 

Unfortunately, data linking UFs and carotenoids are not that consistent. In 2008, an interesting study by Terry et al was performed on more than 82,000 patients and total lycopene intake was not associated with UF risk. On the other hand, the intake of beta-carotene was associated with slightly increased risks of UF diagnosis, but this was restricted only to women who smoke. Thus, authors concluded that their findings did not suggest that carotenoids reduce the risk of diagnosed UFs, and there is only small risk in current smokers [200]. Since lycopene works in part to modulate the synthesis of cell cycle regulating proteins [201] and thus induces an anti-proliferative effect and regulates anti-tumor immunity [202]. Sahin et al. studied the effect of lycopene supplementation on oviduct fibroids in Japanese quails and observed a lower incidence of tumors and dose-dependent reduction of tumor size in the treatment versus control group. The treated Japanese quails also had significantly lower levels of oxidative stress biomarkers, including homocysteine and malondialdehyde, and higher serum amounts of antioxidant vitamins A, C, and E compared to untreated controls [203]. In another in vivo study, Sahin et al. tested the effects of diet derived lycopene by treating Japanese quails with different doses of tomato powder. After one year of supplementation, the treated quails had a significantly decreased number of UFs as compared to control [204]. Tomatoes contain not only lycopene, but also other active nutrient substances, like folate, phenolics, vitamins A and C, and flavonoids [205], which are also known to possess antioxidant activity [206]. An inverse correlation between tomato intake and risks for various tumors, including prostatic, lung, pancreatic, gastric, cervical, and ovarian cancers has been shown [207]. Additional carotenoids in tomatoes, like phytoene and phytofluene, may have an additive effect with lycopene, thus potentiating the antioxidant action. 

Lycopene has a promising potential role in UF management. Lycopene is well tolerated and widely consumed in many global diets. It deserves more research attention to achieve proper dosing, efficacy, and tolerability. 

#### 3.1.15. Collagenase *Clostridium histolyticum *

Collagenase is an enzyme that has an effect on collagen and produced by the bacterium *Clostridium histolyticum* [208]. Since the year 2010, after approval from United States FDA, it has been widely used for fibrotic-dependent disease, e.g., in Dupuytren’s contracture [209]. Since UFs contain a great percentage of abundant and disorganized collagen-rich ECM, this anti-collagen enzyme may serve as an efficient non-hormonal treatment option. Brunengraber et al. assessed the potential utility of this agent as treatment for UFs in human samples collected after hysterectomy. Authors found that the relative percentage of collagen-stained area in UF-injected samples was less than that in both control samples and treated myometrium. The main limitation is the heterogeneity of ECM and collagen which might contribute to varied responses and needs additional studies [209]. Jayes et al. used a highly purified collagenase *Clostridium histolyticum* to explore how it reduces the stiffness of UF tumor. The authors used 3 doses of collagenase. Longer incubation time caused greater reduction in tumor stiffness, shape, and consistency. The authors concluded that the reduction of tissue stiffness is of much importance as it might decrease bulk symptoms and lead to UF volume decrease, probably leading also to pain and bleeding reduction [210]. These promising data brought this agent to the first phases of clinical trials where it was studied in humans.

In a study by Singh et al. [211], 15 women with UFs who had planned surgical treatment were enrolled in a trial to test how collagenase *Clostridium histolyticum* acts in living humans. Authors injected the collagenase into lesions which were later removed during hysterectomy. This was the first study to check the potential safety and tolerability of collagenase injection and change in collagen content was checked. Authors found that treated samples had an average of 21% reduction in density of collagen bundles in comparison to controls. Moreover, the digestion of collagen did not extend the capsule of any lesion. Thus, no adverse drug reactions or adverse event were reported by patients. As for symptoms, patients reported a decrease of pain after drug injection [211]. 

In our opinion, this promising compound is unique in UFs management. Most of the discussed agents are used orally or intravenously while this one is the first to be injected directly to the lesion during different procedures, like in out-patient clinics with the use of ultrasound and long needle, or during surgeries like hysteroscopy and laparoscopy. Of course, this has some advantages and disadvantages as well. The advantage is that this will be a rather selective treatment of individual lesions. The disadvantage is this will be a new, safe form of non-invasive treatment. A new intrauterine system releasing such collagenase would be interesting, but requires further research. 

**Figure 3:**
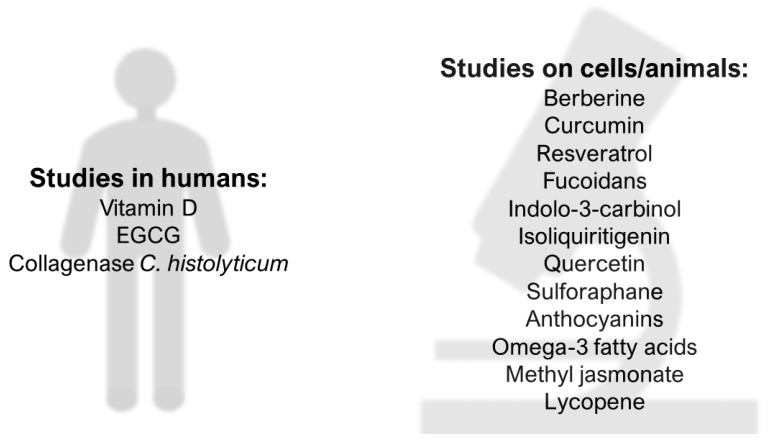
Promising natural anti-UF compounds. Epigallocatechin gallate—EGCG.

We summarize the available compounds in Figure 3.

The most important pathways and effects of described natural compounds are presented in Table 1.

### 3.2. Bioavailability of Natural Compounds

Bioavailability is crucial step to ensure the bio-efficacy of natural compounds. It is a complex process including liberation, absorption, distribution, metabolism, and elimination phases (LADME). Natural compounds need to be bioavailable in order to exert their beneficial effects. Many variables can affect their bioavailability, such as bio-accessibility, transporters, molecular structures, and metabolizing enzymes. Since hydrophilic and lipophilic bioactive compounds have different mechanisms of absorption, the bioavailability of these compounds is challenging. Several technologies have been developed to enhance the bioavailability including structural modifications, nanotechnology, and colloidal systems. Surprisingly, there is little data on factors that affect absorption of natural compounds [212]. 

Fat-soluble vitamin D, ergocalciferol, the form mostly used in supplements and fortified foods, is apparently absorbed with similar efficiency to cholecalciferol (vitamin D3, the main dietary form). Moreover, 25(OH)D, the metabolite produced in the liver, and which can be found in foods, is better absorbed than the non-hydroxy vitamin D forms cholecalciferol and ergocalciferol. Studies also concluded there is insufficient, or even no data, on the effect of type and amount of dietary fiber, vitamin D status, and genetic variation in proteins involved in its intestinal absorption on vitamin D bioavailability [213]. Despite all of its pleiotropic properties, most of EGCG's health-promoting effects are compromised following oral administration due to its poor intestinal permeability and stability [214]; however, its promising effects have attracted increasing interest to scientists in the last decades to improve its bioavailability. For example, folic acid-functionalized nanostructured lipid carriers (NLC) loading EGCG has been recently developed to increase its oral bioavailability [215]. Certain studies highlighted low curcumin bioavailability, but it has been addressed by using higher concentrations of curcumin within nontoxic limits. Moreover, curcumin, in combination with other compounds or as formulations, has shown enhanced bioavailability. Hence, bioavailability is not a problem in the curcumin-mediated treatment of chronic diseases [216]. Additionally, several formulations have been developed and showed better bioavailability mostly attributed to improved solubility, stability, and possibly low first-pass metabolism [217]. Resveratrol is extensively metabolized and rapidly eliminated and therefore it shows a poor bioavailability, in spite it of its lipophilic nature. During the past decade, in order to improve its low aqueous solubility, absorption, membrane transport, and its poor bioavailability, various methodological approaches and different synthetic derivatives have been developed like nanoencapsulation in lipid nanocarriers or liposomes [218]. Studies are still running for better understanding of cruciferous vegetables and sulforaphane bioavailability [219]. The absolute bioavailability of quercetin in humans was estimated at 44.8% and half-life in the range of 11–28 h; this indicates a likely significant increased plasma concentration consequent to continuous supplementation [220]. Lycopene has a low bioavailability rate and appears in the blood circulation incorporated into chylomicrons and other apo-B containing lipoproteins. The recent body of evidence suggests that plasma concentration of lycopene is not only a function of intestinal absorption rate but also lycopene breakdown via enzymatic and oxidative pathways in blood and tissues. Its bioavailability depends on various factors such as food processing or co-ingestion of fat [221,222].

### 3.3. Clinical Application of Natural Compounds in UFs Management

The concept of using complementary and alternative medicine (CAMs) in therapy was established centuries ago. Plants and herbs are key parts of traditional medicine. Additionally, pharmacology was initially based on natural compound before it shifted heavily towards synthetic agents. Still, numerous drugs in use are of natural origin and are heavily studied nowadays, especially in oncology. In their review, Newman and Cragg encouraged further exploration of natural compounds instead of only focusing on synthetic agents. Natural compounds may still serve as efficacious drugs for a multitude of disease indications [223]. Nonetheless, several challenges including poor bioavailability of some dietary phytochemicals, instability associated with pH and/or enzyme-mediated degradation, nonselective effects, and finally the need of non-physiological concentration to cause the clinical effect might explain why some of these promising compounds did not progress beyond laboratories to find their place in clinical practice. However, advances in formulation or agent modification might help [224].

According to the 2012 National Health Interview Survey (NHIS), which included a comprehensive survey on the use of complementary health approaches by Americans, 17.7% of US adults had used a dietary supplement other than vitamins and minerals in the past year [225]. The National Institutes of Health (NIH) is dedicated to highlighting the usage of CAM as a way to encourage more research in this field via the National Center for Complementary and Integrative Health (NCCIH). By the definitions provided by the NCCIH, complementary therapies are used in conjunction with conventional medicine, whereas alternative therapies are used in place of conventional medicine.

In this manuscript, we emphasized potential dietary phytochemicals for UF treatment to be used as complementary or alternative medicines, or both, depending on individual patient preferences and treatment plan. The anti-hormonal effect is common in current anti-UF therapies with adverse effects of hot flashes, loss of bone mineral density, vaginal dryness, and benign endometrial changes [49,55]. Oral GnRH analogs, like elagolix, are combined with add-back therapy to decrease these adverse effects but still some patients might experience difficulties [50]. There are also some concerns about other hormonal agents in this indication, such as UPA, which might be connected with liver toxicity [57]. Furthermore, there are no currently available medical options for UFs in women who wish to conceive or during pregnancy. The desire to avoid these side effects, stress connected with potential surgery, and further fertility plans drive the demand for non-hormonal treatments like naturally derived compounds [11,226]. Although UFs prevalence peaks in the 40s age range, Marsh et al. have shown that UF are present in 15% of asymptomatic women aged 18‒30 using ultrasound [227]. Thus, women at higher risk of developing UFs, including those with family history, black race, or delayed childbearing may benefit from naturally-derived compounds to slow or halt UF proliferation or prevent UF development [7,61]. Several reviewed compounds in this article show promise as natural CAM therapies for women with UFs since they affect numerus important pathways involved in UFs, e.g., inflammation, growth factors, angiogenesis, fibrosis, and cell proliferation (cycle arrest or apoptosis) [224]. 

The link between hypovitaminosis D and incidence of many diseases is clear. The American College of Obstetricians and Gynecologists (ACOG) recommends 600 international units (IU) of vitamin D daily for pregnant and lactating women [228]. Most prenatal vitamins typically contain 400 IU of vitamin D per tablet. When vitamin D deficiency is identified during pregnancy, ACOG states that 1000–2000 IU of vitamin D per day is safe. Although ACOG noted that higher dose regimens used for the treatment of vitamin D deficiency have not been studied during pregnancy, it also states that some experts support supplemental vitamin D in dosages up to 4000 IU per day during pregnancy or lactation [228]. Target 25(OH)D serum levels should be about 30-50 ng/ml [62,229]. Research on the impact of vitamin D treatment for UF in humans should use these doses as guidelines.

Tea is one of the most commonly consumed beverages worldwide. Green tea, although most commonly consumed in East Asia, is increasingly popular in the United States due to the many reported health benefits and as a source of caffeine. It is available in brewed drinks as well as powders, oral supplements, and is an additive in food products from energy drinks to ice cream. The current recommendations on caffeine consumption in pregnancy and women seeking conception state that moderate use, less than 200 mg of caffeine per day, appears to be safe [230]. Pregnancy outcomes thought to be associated with large caffeine use include miscarriage, preterm birth, and intrauterine growth restriction. Of these, moderate caffeine consumption does not appear to be a contributing factor based on the literature available to date [230]. Polyphenols, such as EGCG, in green tea may play a role in mammalian metabolism [231]. Concerns regarding increased polyphenol intake during pregnancy have focused on maternal metabolism during pregnancy and intrauterine growth restriction of offspring. Whether these outcomes are associated with green tea use in humans remains to be determined [231]. Animal studies have shown possible decrease in maternal diabetes-associated congenital malformations, such as neural tube defects due to EGCG exposure during pregnancy [232]. However, given the widespread consumption of green tea throughout the world, including during women’s reproductive years, the recommendation for moderate consumption in the brewed form appears reasonable. Eight ounces of brewed tea averages about 50 mg of caffeine [230]. Therefore 1–4 servings daily would keep one within the recommended range for caffeine consumption. Regarding EGCG supplementation, future studies should aim to provide dosing similar to what is found in 8–32 ounces (1–4 servings) of green tea daily to examine the safety profile of a commonly consumed and recommended dose. The idea of combining vitamin D with EGCG is also of potential interest [81].

Berberine may be less commonly used in the United States but is reportedly widely used in Eastern medicine therapies. In China, a total of 800 million 0.1 mg tablets were consumed in 2000, increasing to 5.9 billion in 2013 [233]. In Western medicine, variation in product quality is an issue with pharmacologic use, which could pose a barrier to berberine's safety and effectiveness in clinical practice [234]. Further, berberine consumption in the United States is often associated with ingestion of goldenseal or yellow root, a plant native to North America. Pregnant or breastfeeding women should not use goldenseal, and it should not be given to infants due to hepatotoxicity [235]. Berberine has been studied in reproductive aged women with obesity and polycystic ovarian syndrome. Lin et al. investigated the effect of consuming 0.4 g berberine three times daily for four months in 102 anovulatory Chinese women with polycystic ovarian syndrome [236]. All women received the treatment and were analyzed according to normal weight versus overweight/obese. The treatment was reported to be well-tolerated in all women and only one woman reported gastrointestinal upset. Although this prospective cohort study showed participants improvements in ovulation and regulation of menses, all women were advised to use contraception during the study period given the concern of potential teratogenicity. Later studies have included berberine utility for women seeking conception as an ovulation induction agent like clomiphene citrate or letrozole [233]. More laboratory and human clinical trials are necessary to better understand the safety profile and efficacy of berberine for UF treatment. 

Traditionally, studies showed that the risk of UF development was inversely associated with the intake of vegetables and fruit and increased with beef and ham consumption [237]. In 2011, Wise et al. found that UFs risk was not associated with dietary intake of vitamins C and E, folate, fiber, and carotenoids [188].

### 3.4. Future Direction

**Figure 4:**
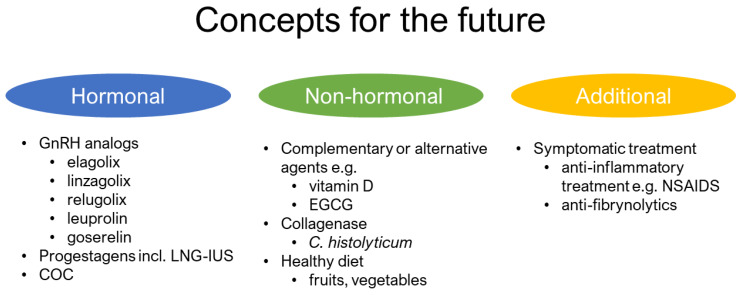
Current concepts on the use of natural anti-UF compounds. combined oral contraception—COC; epigallocatechin gallate—EGCG; gonadotropin-releasing hormone—GnRH; levonorgestrel-releasing intrauterine system—LNG-IUS; nonsteroidal anti-inflammatory drugs—NSAIDS.

There is an urgent need to find cost-effective, safe, and effective drugs for the prevention and treatment of UFs. Vitamin D is a natural supplement with pleiotropic action and the potential to prevent UF development and growth and deserves great attention and further investigation [63,84]. Similarly, EGCG also showed good laboratory and clinical data. Further reports are necessary to prove the efficacy of EGCG supplementation in women. The concept of combining their therapeutic effects could be additive and translates into an effective clinical therapy [224] especially given their good safety profiles. Further combinations of CAMs should be also considered against UFs. We might also search for less popular compounds, e.g., Chinese formulas, which were found to adjust the influence of mifepristone in reducing the volume of UFs and improve the symptoms of dysmenorrhea [238]. Studies about potential synergism between new oral GnRH analogs like elagolix, relugolix, and linzagolix and presented CAMs might also show interesting results. The concept of available possibilities in co-drugs is described in Figure 4, were we have included the summarized data from this manuscript with the current and emerging therapies available in UFs as presented by Chwalisz et and Taylor [239].

Additionally, considering synthetic analogs of natural compounds might be worth pursuing as well to overcome any bioavailability or dosing challenges. This can be shown for vitamin D and curcumin. Halder et al found that vitamin D analog paricalcitol exhibited more UFs size reduction compared to active vitamin D. Authors suggested that paricalcitol may be a potential candidate for effective, safe option in UFs [75]. Likewise, potent curcumin analog EF24 killed cells and inhibited TNF-α-induced-inflammation pathways with approximately ten fold stronger effect than natural curcumin [240]; however, there is no data about utility of this compound in UFs. 

## 4. Conclusions

UFs remain a significant health issue for many women, and they disproportionately impact women of color. As new pharmaceutic medical options emerge, natural compounds should also be considered as potential options for UF management and prevention. For example, vitamin D or EGCG should be tested in human clinical trials to establish its safety and clinical effect. Other compounds are promising in in vitro or in vivo models. However, they need further research to determine mechanism of action and safety profile in reproductive aged women. The concept of combined patient-tailored therapies is valid for further investigation and can be combined with the known agents to achieve better results.

## Figures and Tables

**Table 1:** Most important pathways and effects of natural anti-UF compounds.

Compound	Molecular Target
Vitamin D	MMPs inhibition Catechol-O-methyltransferase suppression TGF-β induced ECM production inhibition Wnt/β-catenin pathway inhibition Steroid receptor expression decrease Anti-inflammatory effect Apoptosis induction/proliferation inhibition
EGCG	MMPs inhibition Catechol-O-methyltransferase suppression Anti-inflammatory effect Apoptosis induction/proliferation inhibition BMP2 expression upregulation
Berberine	Cyclooxygenase 2 inhibition Anti-inflammatory effect PTTG1 inhibition Apoptosis induction
Curcumin	PPARγ activation TGF-β induced ECM production inhibition Apoptosis induction Anti-inflammatory effect
Resveratrol	MMPs inhibition ECM production inhibition Apoptosis induction
Fucoidan	Epithelial–mesenchymal transition inhibition ECM production inhibition Wnt/β-catenin pathway inhibition
Indolo-3-carbinol	ECM production inhibition Anti-inflammatory effect Apoptosis induction
Isoliquiritigenin	MMPs inhibition ECM production inhibition Anti-inflammatory effect Apoptosis induction
Quercetin	Effect on steroid receptors TGF-β induced ECM production inhibition Anti-inflammatory effect
Sulforaphane	Effect on TGF-β pathway Anti-inflammatory effect
Anthocyanins	ECM production inhibition Anti-inflammatory effect
Omega-3 fatty acids	Anti-inflammatory effect Lipid profile modulation
Methyl jasmonate	Enhancer of zeste homolog 2 inhibition ECM production inhibition Wnt/β-catenin pathway inhibition Apoptosis induction
Lycopene	Immunomodulation Apoptosis induction
Collagenase *C. histolyticum*	ECM degradation

Bone morphogenetic protein 2–BMP2; extracellular matrix–ECM; metalloproteinases–MMPs; peroxisome proliferator-activated receptor γ–PPARγ; pituitary tumor transforming gene 1 protein–PTTG1; transforming growth factor beta–TGF-β; wingless-type signaling pathway–Wnt.
